# High-Accuracy reduction of comminuted diaphyseal femur fractures using on-site 3-D-printed guides

**DOI:** 10.1007/s00068-025-02970-z

**Published:** 2025-09-23

**Authors:** Sophie C. Eberlein, Samuel F. Schaible, Frank M. Klenke, Andreas Hecker

**Affiliations:** 1 Department of Orthopedic Surgery and Traumatology, University Hospital Bern, Inselspital, University of Bern, Bern, Switzerland; 2 Gelenkzentrum Bern AG, Salem Spital, Hirslanden Group, CH-3013 Bern, Switzerland; 3 ArthroClinic Bern Schweiz, Lindenhofspital, CH-3012 Bern, Switzerland

**Keywords:** Femoral shaft fracture, 3-D printing, Patient-specific reduction guide, Rotational malalignment, Cadaveric study, Orthopedic trauma

## Abstract

**Purpose:**

Persistent malalignment—especially rotational error—after fixation of comminuted femoral shaft fractures compromises function and often necessitates revision surgery. This study evaluated whether point-of-care, patient-specific three-dimensional printed reduction guides can reproducibly restore native femoral length, coronal–sagittal alignment and axial rotation in a cadaveric model simulating highly comminuted shaft fractures.

**Methods:**

Ten freshfrozen human legs were CTscanned, virtually reduced, and fitted with patientspecific guides printed in a sterilizable medical resin. A 2 cm midshaft segment was resected to simulate a comminuted fracture. Each construct was reduced with the guides and stabilized with a locking compression plate. Postoperative CT volumes were superimposed to the preoperative plan to quantify absolute deviations in length, coronal (varus/valgus), sagittal (procurvatum/recurvatum), and axial rotation.

**Results:**

All reductions were completed without technical difficulty. Mean ± SD absolute errors were 1.50 ± 1.08 mm in length, 0.92 ± 0.41° in the coronal plane, 1.33 ± 1.34° in the sagittal plane, and 4.33 ± 1.88° in rotation—well within acceptable clinical limits.

**Conclusion:**

On‑site printed, patient‑specific guides achieved high accuracy in a comminuted femoral fracture model. Clinical studies are required to determine whether this accuracy translates into improved patient outcomes.

## Introduction

Comminuted diaphyseal fractures of the femur present substantial challenges for maintaining anatomical alignment, especially rotational orientation. Rotational malalignment following femoral shaft fracture fixation remains a significant concern, with reported rates varying from 2.3 to 35% in the transverse plane [1]. Recent clinical studies demonstrate more specific deformity patterns: Orfeuvre et al. found stereoradiographic rotational malrotation ≥ 15° in 29.2% of patients following antegrade nailing, with mean rotational anteversion of 18.5 ± 13.8° [2]. A systematic review by Jankowski et al. reported mean absolute deviations for intramedullary nailing as: 4.33 ± 1.88° rotational malalignment (transverse plane), 0.92 ± 0.41° varus-valgus angulation (coronal plane), 1.33 ± 1.34° flexion-extension angulation (sagittal plane), and 1.50 ± 1.08 mm length discrepancy [3]. These deformities, particularly rotational errors exceeding 10–15°, can lead to altered biomechanics and functional impairment [1–4]. Moreover, revision surgery specifically for symptomatic rotational malalignment occurs in approximately 2.1% of patients following intramedullary nailing [2]. In an effort to address these difficulties, recent work has focused on three-dimensional (3D) printing technologies that can create patient-specific reduction guides aimed at improving intraoperative accuracy and limiting postoperative deviations [5]. Early clinical and preclinical data suggest that such guides can help surgeons achieve reductions that closely match preoperative plans, potentially leading to better functional scores and diminished reliance on fluoroscopic imaging [6, 7]. However, questions remain regarding the reproducibility of these findings in a setting that more closely approximates human clinical anatomy, as well as the broader cost implications of integrating 3D printing into routine orthopedic trauma care [6, 8].

This study evaluated point-of-care 3D-printed reduction guides in ten human cadaveric femora with simulated comminuted diaphyseal fractures. The primary objective was to determine whether on-site manufacturing of patient-specific guides can achieve accurate restoration of length, rotation, and angular alignment. We hypothesized that residual deviations would remain reliably within clinically acceptable thresholds in a real-world context.

## Methods

**Fig. 1:**
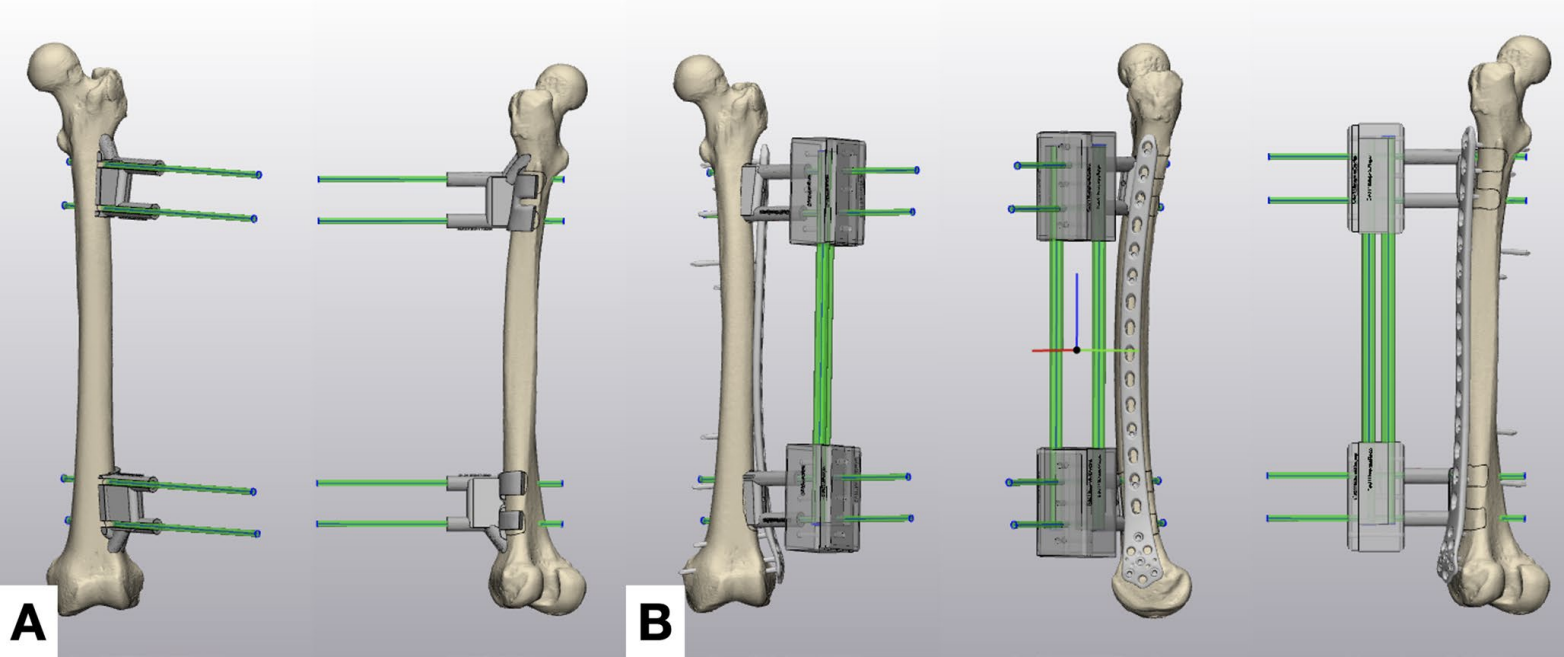
Custom reduction guides. **A**, anterior (left) and posterior (right) renderings of the firststage guide show threepoint metaphyseal bases that lock onto the proximal and distal femur, establishing formfitting registration via “feet” for accurate placement of Schanz screws. **B**, anteroposterior, lateral, and posteroanterior rendered views of the secondstage bridging guide demonstrate boxshaped seats that lock the reduction while engagement with the previously inserted Schanz screws ensures correct orientation, leaving the lateral cortex clear for lockingplate fixation

Ten freshfrozen human cadaveric lower limbs without preexisting deformity were acquired through an accredited anatomical donation program. Each limb underwent highresolution computed tomography (slice thickness 0.5 mm) on a SOMATOM X.cite scanner (Siemens Healthcare, Erlangen, Germany). DICOM data were imported into Mimics 25.0 (Materialise, Leuven, Belgium) for segmentation and threedimensional reconstruction of the femur. Patientspecific reduction guides were engineered in 3matic 16.0 (Materialise). Two sequential guides were produced. The firststage guide was formfitting, relying on a tripod cortical footprint that provided intrinsic stability and ensured precise registration for Schanzscrew insertion. The secondstage bridging guide incorporated an 8 mm cylindrical channel that accommodated carbonfiber rods (Stryker, Kalamazoo, MI, USA), rigidly coupling the fragments; to preserve the lateral corridor for definitive plate fixation, this guide omitted metaphyseal bases but achieved accurate positioning on the femur by indexing onto the previously inserted Schanz screws (Fig. 1).

All guides were additively manufactured in BioMed Amber resin (Formlabs, Somerville, MA, USA) with a Form 3B printer from the same manufacturer. Postprocessing comprised a 20 min isopropanol wash, 30 min of forcedair drying at 50 °C, and 60 min of ultraviolet curing. Once cured, residual support struts were removed with sidecutters, taking care not to abrade the formfitting bonecontact surfaces. Guides were steamsterilised in an autoclave at 134 °C for 18 min (MMM Group, Munich, Germany).

**Fig. 2:**
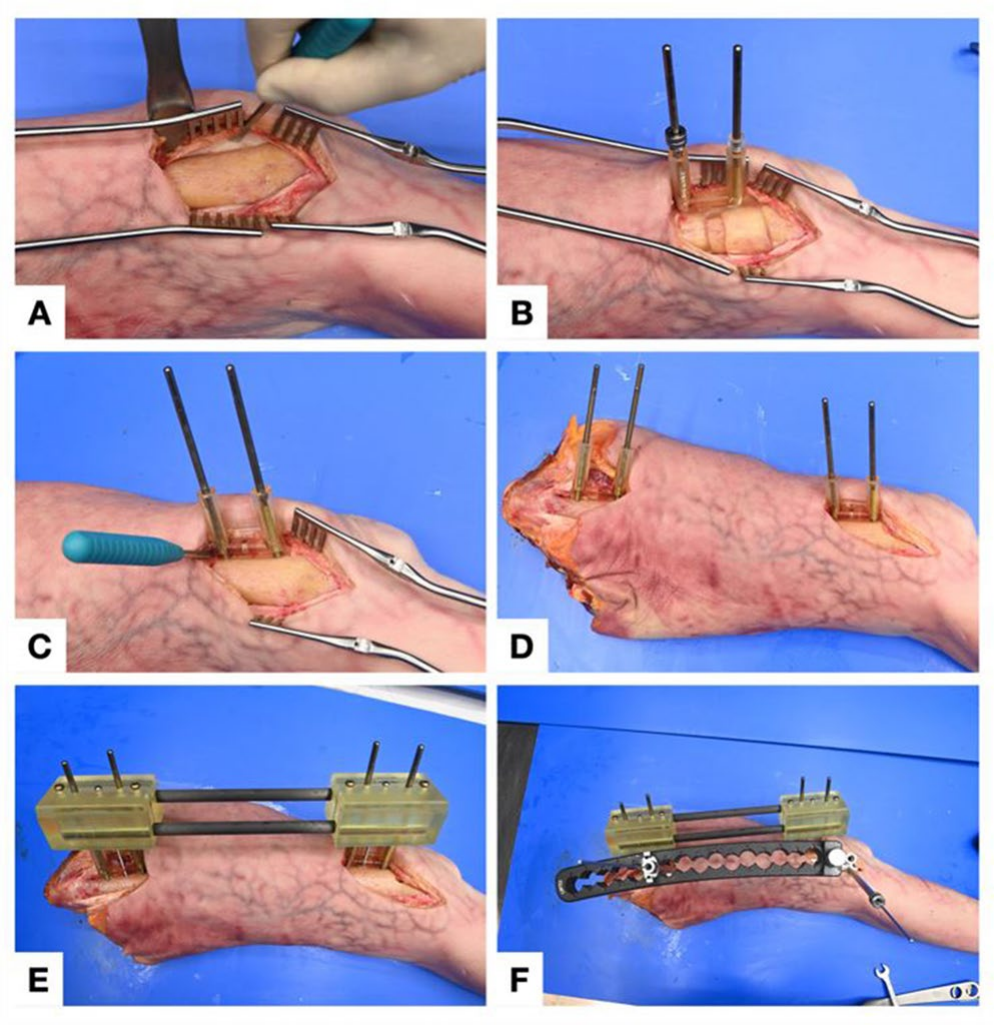
Step-by-step application of the 3D-printed reduction guide system for a simulated diaphyseal fracture of the femur. **A**: Minimally invasive approach to the distal lateral femur and periosteal elevation with the raspartorium. **B**: First-step guides secured with 4-mm Schanz pins on the distal segment. **C**, **D**: Second-step connection guides with a cavity for plate placement slid over the remaining pins **E**: Assembly of the bridging reduction guide with carbon-fiber rods between the proximal and distal constructs. **F**: Final fixation with a 4.5/5.0-mm locking compression plate before guide removal

To simulate a comminuted fracture devoid of intrinsic bony reference, a 2 cm diaphyseal segment was resected though a small separate medial incision. Guide placement took place through minimally invasive proximal and distal approach to the lateral femur. Periosteal elevation was limited to the areas required for guide application (Fig. 2A). The proximal and distal firststep guides were seated and fixed with 4 mm Schanz pins (Stryker, Kalamazoo, MI, USA; Fig. 2B). Sleeves around the Schanz pins enabled easy removal of the guide. Secondstep connection guides were then slid over these pins. A posterior cavity in the guides allowed plate placement on the lateral femur (Fig. 2C–D). A bridging reduction guide, assembled with two carbonfiber rods, rigidly coupled the proximal and distal constructs, thereby enforcing the preplanned alignment (Fig. 2E). Definitive stabilization was achieved with a standard anatomical 4.5/5.0 mm locking compression plate (DePuy Synthes, Solothurn, Switzerland; Fig. 2F), after which all guides and pins were removed.

**Fig. 3:**
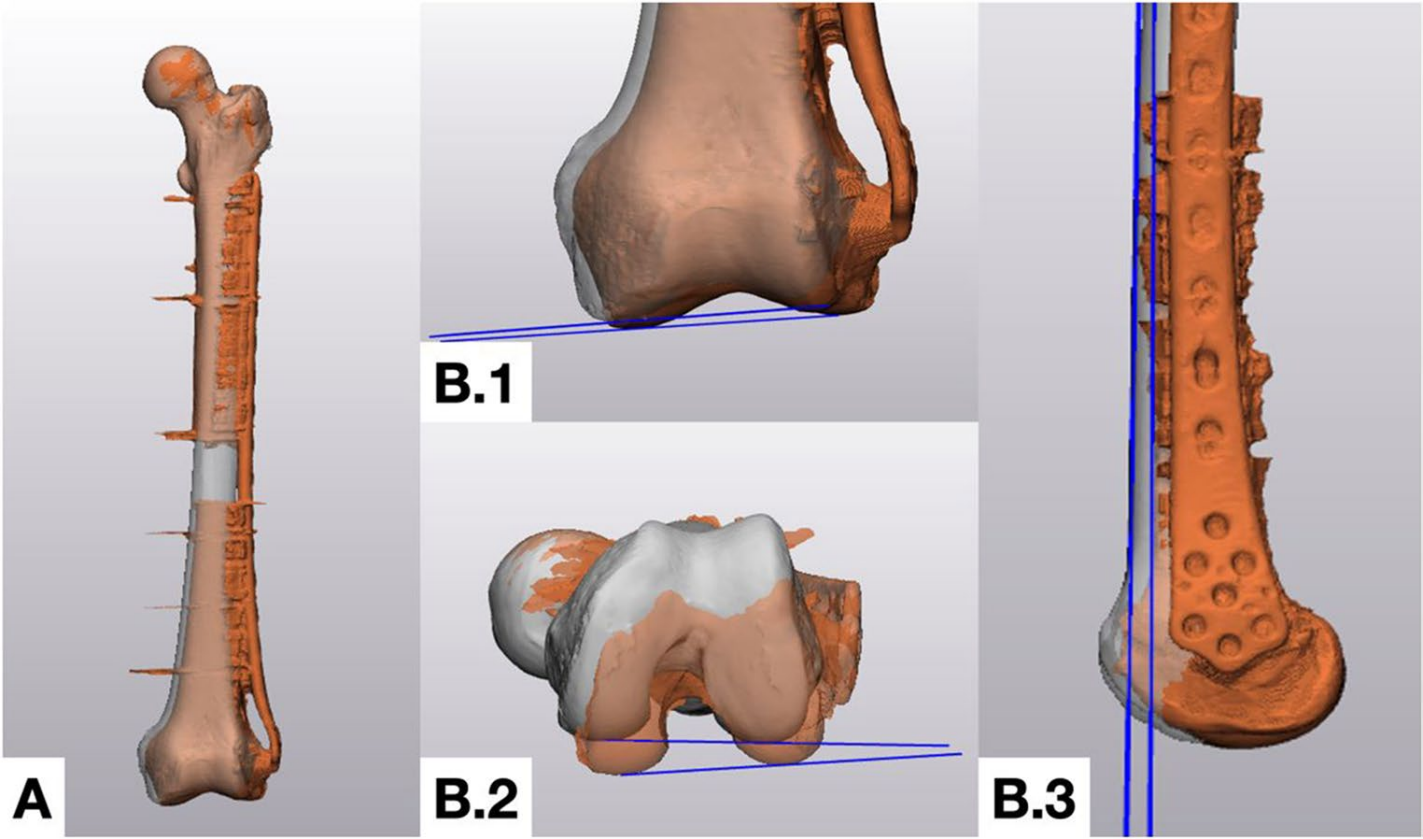
Postoperative measurement workflow after superimposition of original and post-operative alignment. **A**, threedimensional reconstruction of a representative femur after plate fixation demonstrates restoration of the resected diaphyseal segment. B.1, coronal view of the distal femur showing the varus–valgus measurement plane (blue) tangential to the inferior condylar border. B.2, axial (inferior) view illustrating the rotational measurement plane (blue) drawn along the posterior condylar cortex. B.3, sagittal view depicting length assessment from the proximal overlay region to the distal condyles, while procurvatum–recurvatum is measured with a blue line tangent to the anterior diaphyseal cortex. Postoperative models were registered to the preoperative plan, and all deviations were calculated with the proximal fragment fixed as the reference

Each specimen then underwent repeat computed tomography using the identical acquisition protocol. The postoperative volume was rigidly registered to the preoperative plan in 3matic 16.0. To accurately assess deviations, the Materialise 3-matic software allows registration of two similar 3D models to each other (global registration, min. 200 iterations). Alignment was locked to the proximal segment—using the femoral head and greater trochanter as overlay landmarks—and all deviations were quantified in the distal segment (Fig. 3A). Length was measured between reproducible metaphyseal landmarks. Varus–valgus deviation was calculated from a plane tangential to the inferior condylar border, and procurvatum–recurvatum (flexion–extension) was derived from a line tangent to the anterior cortical surface of the diaphysis. Axial rotation was determined from a plane tangential to the posterior condylar cortex. The posterior condylar line was selected as our reference due to its clear visualization on CT and established use in measuring femoral anteversion [1] (Fig. 3B.1–B3).

**Fig. 4:**
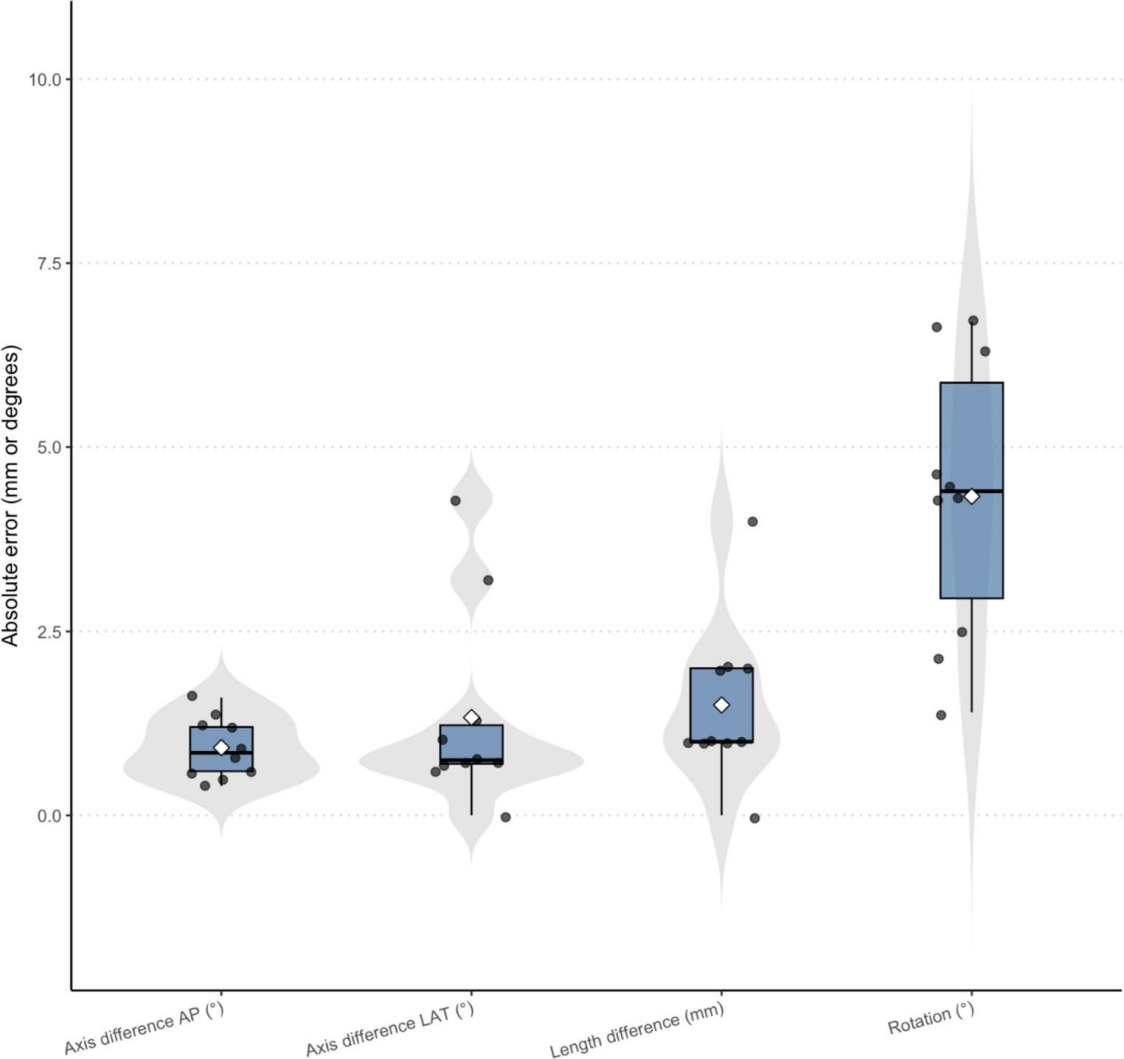
Absolute deviations between planned and achieved femoral alignment (*n* = 10). For each metric—length, coronal (varus/valgus), sagittal (procurvatum/recurvatum), and axial rotation—individual specimens are shown as jittered dots superimposed on violin and box plots; diamonds indicate group means

Statistical analyses were performed with R version 4.2.3 (R Foundation for Statistical Computing, Vienna, Austria). Data normality was assessed with the Shapiro–Wilk test, and results are presented as means ± standard deviations. (Fig. 4)

## Results

Ten freshfrozen femora were studied. All guides printed and seated as planned, and each reduction and plate fixation were completed without difficulty. The ShapiroWilk test showed normal distribution for every variable except absolute length error and lateralplane angulation. The mean absolute error (± SD) was 1.5 ± 1.1 mm for length, 0.9 ± 0.4° for coronal angulation, 1.3 ± 1.3° for sagittal angulation, and 4.3 ± 1.9° for axial rotation. Residual deformity showed a consistent bias toward internal rotation and flexion, each recorded in seven of the ten specimens (Table 1).

**Table 1:** Descriptive statistics of reduction accuracy for 10 femora

Parameter	Abs Mean ± SD	Min-Max	Directional Tendency	%
Length difference (mm)	1.50 ± 1.08	0.00‑4.00	Shortening	50%
AP axis difference (°)	0.92 ± 0.41	0.40‑1.60	Valgus	70%
Lateral axis difference (°)	1.33 ± 1.34	0.00‑4.30	Flexion	70%
Rotation (°)	4.33 ± 1.88	1.40‑6.70	Internal rotation	70%

Values reported as absolute deviations from zero (planned reduction)

## Discussion

This cadaver study shows that pointofcare 3Dprinted reduction guides restore femoral shaft alignment with mean residual errors of 1.5 mm in length, 0.9° in the coronal plane, 1.3° in the sagittal plane, and 4.3° in axial rotation—all well inside the strictest recommendations for diaphyseal fractures. Although the definition of a “malrotated” femur is contentious, permissible torsional deviations in the literature range from 10° to 30° [9–11]. 

Even the lower end of that interval is clinically relevant: torsional mismatch of ≥ 10° relative to the opposite limb can produce anterior knee pain and patellofemoral dysfunction [12], and larger errors accelerate hip and knee osteoarthritis [13]. Angular errors beyond 5° and length discrepancies greater than 10 mm likewise correlate with symptomatic malunion and premature joint degeneration [14, 15]. Against this backdrop, the 4.3° rotational error and low singlemillimeter linear as well as sub to lowdegree angular deviations achieved here constitute a meaningful advance over conventional reduction, which continues to yield femoral malrotation rates approaching50% [16]. These results closely parallel the accuracy we reported in a porcine feasibility model that used the same pointofcare workflow [5].

Our guide‑based technique achieves rotational errors within the range reported for comparable applications (3.5°–8.3° in forearm osteotomies and < 2° angular deviation in patient‑specific high‑tibial osteotomy) [17, 18]. While intramedullary nailing remains the gold standard for diaphyseal femur fractures, certain clinical scenarios may favor plate fixation. In this study, we utilized plate fixation with patient-specific guides to demonstrate accurate reduction. The principle of guide-based reduction could potentially be adapted for use with intramedullary nailing, though this would require modification of the guide design and surgical workflow. The implant is advanced through a submuscular tunnel so the fracture zone remains untouched; periosteal stripping is therefore minimal, preserving cortical perfusion and the biologic milieu essential for union [18]. In addition, all guide contact surfaces are located outside the fracture line, where vascularity is most vulnerable, affording further protection of local blood supply [19]. While this cadaveric study demonstrates mechanical accuracy, clinical benefits including operative efficiency and patient outcomes require prospective validation.

Alternative techniques for optimizing reduction include intraoperative clinical examination, radiographic rulers, and perfect lateral imaging. Each method has limitations: clinical examination is challenging with bilateral injuries, and fluoroscopic assessment methods show mean rotational errors of 6.0° to 8.5°, with the lesser trochanter profile method resulting in > 15° malrotation in 20% of cases compared to only 2.5% with the true lateral method and 5% with the neck-horizontal angle method [20].

Several limitations must be acknowledged. First, the cadaveric model lacks active soft tissue tension, which can challenge guide seating in vivo; however, the residual discrepancies we recorded are smaller than the side‑to‑side intraindividual variation seen in healthy adults [20]. Second, comminution was simulated with a segmental defect; future work should address complex multiplanar fractures with potential fragment dislocation that make closed reduction impossible. Third, our minimally invasive approaches required 3–4 cm incisions at the proximal and distal metaphyseal regions. Fourth, we did not formally test intentional guide malplacement; the potential for mis‑seating and its impact on accuracy warrant investigation. Finally, while mechanical accuracy is necessary, its influence on functional outcome will require prospective clinical confirmation.

In summary, patient‑specific 3‑D reduction guides produced femoral reductions that surpassed the most conservative clinical thresholds for length, axis, and rotation. These findings, together with our prior porcine data, demonstrate the technical feasibility and biomechanical accuracy of in‑house printed guides for achieving anatomic reduction in complex femoral shaft fractures, where traditional plate fixation techniques remain prone to malalignment. Clinical validation is warranted to determine whether this accuracy translates to improved patient outcomes.

## Conclusion

Three‑dimensionally printed, patient‑specific reduction guides restored femoral shaft alignment to within sub‑millimeter and sub‑degree tolerances, well inside conservative clinical standards. This cadaveric feasibility study demonstrates mechanical accuracy; prospective clinical studies are required to determine effects on patient outcomes, revision rates, operative efficiency, and resource use.

## Data Availability

De-identified CT datasets and planning files that support the findings are available from the corresponding author upon reasonable request.
